# A Sustained Partnership between a Haitian Children’s Hospital and North American Academic Medical Centers

**DOI:** 10.3389/fpubh.2017.00122

**Published:** 2017-05-30

**Authors:** Michael P. Koster, Jackson H. Williams, Jacqueline Gautier, Renee Alce, Bernard E. Trappey

**Affiliations:** ^1^Department of Pediatrics, Brown University Alpert Medical School, Providence, RI, United States; ^2^Department of Pediatrics, East Tennessee State University Quillen College of Medicine, Johnson City, TN, United States; ^3^Pediatrics, Hôpital Saint Damien-Nos Petits Frères et Soeurs, Tabarre, Haiti; ^4^Department of Medicine and Pediatrics, University of Minnesota Medical School, Minneapolis, MN, United States

**Keywords:** global health, collaborative, Haiti, pediatrics, academic medical center, medical education, training, capacity building

## Abstract

Global health initiatives from academic medical centers have rapidly proliferated over the last decade. This paper endeavors to describe our 5-year experience as an academic medical collaborative supporting healthcare delivery, medical training, and research at Hôpital Saint Damien-Nos Petits Frères et Soeurs, the only freestanding children’s hospital in Haiti. Descriptions of the history and current activities of our academic medical collaborative, its partnership and communication structure, its evolution to fill the expressed needs of our host site, its funding mechanisms, and its challenges and opportunities for the future are included.

## Introduction

According to the World Bank, Haiti is the most financially constrained nation in the Americas and one of the most economically depressed nations in the world. Over 2.5 million Haitians are under the national “extreme poverty line” living on less than United States (US) $1.23 per day (1, 2). Delivery of adequate pediatric healthcare remains a challenge in Haiti and was greatly complicated by the devastating earthquake of January 12, 2010. After the earthquake, the under-5-year-old mortality rate spiked to over 150 deaths per 1,000 children (1, 2). The country is now making strides toward recovery, but training an adequate number of physicians—and specifically pediatricians—remains a challenge. It is estimated that there are as few as four health professionals per 10,000 people (1, 2). Agencies like USAID have identified human resources for health as a key challenge for Haiti (3). Given the magnitude of health disparity and lack of capacity building for human resources, the process of developing a sustainable, long-term partnership with a children’s hospital in Haiti is described.

## History of Hôpital Saint Damien-Nos Petits Frères et Soeurs (HSD) and the Saint Damien Academic Medical Collaborative (SDC)

Nuestros Pequeños Hermanos International, “Our Little Brothers and Sisters,” is a Catholic relief charity established in Mexico since the 1950s. The organization expanded to Haiti in 1988 and founded the HSD in 1991. HSD began as a pediatric hospice facility for children dying of HIV/AIDS, but soon developed into a full-service children’s hospital. The hospital currently provides over 50,000 services per year to the children of Haiti and provides the only pediatric oncology treatment center in the country. A high-risk maternity unit was also developed and coupled with a neonatal intensive care unit, one of only six in the country. After the earthquake of 2010, HSD was one of the few hospitals near Port-au-Prince that was left largely intact. As a result, medical and surgical teams from around the world descended on HSD as part of the relief effort. This resulted in overflowing rooms, and hallways with courtyards converted into temporary patient wards and housing for staff and international volunteers. After emergency relief efforts wound down, a group of faculty and resident physicians from several North American (NA) academic medical centers (AMCs) continued to make regular trips to support HSD. Executive leadership at HSD asked for one site to lead coordination efforts. The result was the SDC, the beginnings of which were several phone conversations and decisions about when groups would be traveling so as not to overlap and cause a drain on the HSD system (4). In 2011, monthly conference calls ensued and minutes were kept in order for teams to have up-to-date information and synchronize efforts. Conference calls became more robust and eventually lead to videoconferences that have standing agenda items beginning with an update from HSD. All members have a dedicated email and access to a virtual drive where conference call minutes and other shared items can be found. The institutions and their year of their matriculation are listed in Table 1.

**Table 1:** **Saint Damien Academic Medical Collaborative institutions and year of enrollment**.

Institution	Academic affiliation	Year joined
Akron Children’s Hospital	Northeast Ohio Medical University	2012
Children’s Hospital of the King’s Daughters	Eastern Virginia Medical School	2012
Hasbro Children’s Hospital	Brown University	2012
Rainbow Babies and Children’s Hospital	Case Western University	2012
UMass Memorial Children’s Medical Center	University of Massachusetts	2012
Masonic Children’s Hospital	University of Minnesota	2012
Baystate Children’s Hospital	Tufts University	2014
Omaha Children’s Hospital and Medical Center	University of Nebraska	2014
Dell Children’s Medical Center	University of Texas Dell Medical School	2015
Niswonger Children’s Hospital	Quillen College of Medicine, East Tennessee State University	2016
Montreal Children’s Hospital	McGill University	2016

The SDC determined fundraising was a priority, and in 2012, a conference and fundraiser took place at the primary coordinating site. This inaugural event led to other partner institutions leading similar pediatric global health conferences and fundraisers for HSD each year, with $94,640 raised over four events. Most recently in 2016, in a matched fundraising drive, the SDC raised $50,000 resulting in $100,000 for HSD. The SDC’s activities are largely funded, independent of its member institutions, through private fundraising events and grants. Each member institution pays a small annual membership fee which helps fund a coordinator and chief resident position at HSD, both of whom support the visiting members of the collaborative and the Haitian Pediatric residency program.

At each conference, there has been an annual meeting of the SDC, with representation from the HSD leadership, and often with the introduction of new member institutions. The focus has always been on the current needs of HSD and the strategic plans for how SDC can partner to help meet these needs during the next year. Each partner site is able to share integral face-to-face time with HSD administration and clinicians, hearing their goals and expressed needs.

In the fall of 2013, HSD started a pediatric residency program with curriculum created by Haitian staff and faculty. Importantly, while this was a local initiative targeting their own identified needs, the breadth of the SDC provided an educationally supportive backdrop to the residency. The Haitian pediatric residents rotate at two hospitals: HSD and Hôpital Bernard Mevs (HBM). The combination of HSD’s large pediatric patient volume and the Pediatric Intensive Care Unit at HBM gives the residency a blend of experience unique in Haiti. Since 2010, visiting faculty and residents have continued to regularly rotate at HSD, but as a result of SDC efforts, more structure and coordination now exists. Supporting the HSD-HBM residency program was identified as the primary mission of the SDC, solidifying its commitment to improving human resource capacity building for pediatrics in Haiti. A tabulation of faculty and trainee visits from 2010 to 2016 can be found in Table 2.

**Table 2:** **Visiting participants by role**.

Position	Total individuals	Total visits
Resident	53	67
Fellow	15	16
Faculty	43	102
Medical student	7	7
Other staff	6	6
Total visits	124	198

## Nuts and Bolts of the SDC

The overarching goal of the SDC is partnership with HSD in a mutually rewarding and long-term collaboration that seeks to support the academic mission: enhance delivery of care, support medical education, and develop research capacity.

### Clinical Care

Initially, providing clinical care was the primary objective of most partner sites. Looking to lend a helping hand, the NA institution’s global health faculty visited HSD to provide direct patient care. The early visits focused on relief efforts, but also sought to undertake needs assessments to determine if HSD would be an appropriate long-term partner site. Many of the backing AMCs had significant experience creating partnerships and global health programs. Initially, much of the relief work focused on seeing patients in the emergency room, helping with triage, and providing relief for overwhelmed Haitian physicians dealing with the earthquake aftermath. The model for visiting teams in Haiti is to have an attending present for 1 week and visiting senior residents (typically two) remain for 1 month. This process allows for better acculturation of visiting residents over time, and for a seasoned attending to help with orientation and culture shock in the first week, relieving busy Haitian faculty from this burden. Over time, groups have also worked on the inpatient wards and outpatient areas. Teams participate in hospital-wide rounds twice weekly with a senior Haitian attending physician, and since 2013, with the introduction of the HSD-HBM residency program, morning report and resident noon conference have become commonplace. The strength of an AMC collaborative is that each site can send residents resulting in several months of continuous presence. This also allows HSD staff to also acclimate to visiting physician presence. HSD administration report that Haitian staff comment that having visiting doctors encourages their own self-directed learning. Cardiac surgery is a specific example of collaborative subspecialized care with one partner site. This partner hospital linked with other Non-Government Organizations interested in providing cardiac screening and surgery. Over the last 5 years, nine children have come to the US for cardiac surgery. The focus of this program has now shifted to performing these cases at HSD, and thus incorporating surgical training for Haitian staff. Through 2016, four separate training programs have been conducted, which included cardiac surgery for 36 Haitian children at HSD. Two examples of remote care include review of radiological studies from pediatric radiologists at partner sites and a real-time case consultation via email to allow subspecialists from partner sites to weigh in on complicated clinical cases as part of the ongoing support of the HSD-HBM residency program.

### Education

Education is the foundation of quality medical care, balancing the pillars of healthcare delivery and research. All visiting partner sites that rotate through HSD participate in the education of trainees and staff. The educational elements consist of grand rounds lectures, presentations at the bi-annual National Haitian Pediatric Congress, dissemination of research findings, development of case-based learning modules, hands-on learning of life support and cardiopulmonary resuscitation [i.e., pediatric advanced life support (PALS)], suturing, other procedural workshops including chest tube placement, and bedside ultrasound techniques. When each partner site sends a group to HSD, the attending physician, often a subspecialist, will present grand rounds to the entire hospital staff, including nurses and administrators. HSD has a fully functional conference room, providing a forum for internationally recognized lecturers year round. Since the inception of the HSD-HBM residency program, there is also a daily noon conference, during which the visiting resident rotators can also present topics. An additional area of focus for one partner had been to develop case-based modules, focusing more on problem-based learning and clinical reasoning skills. This was an expressed need from both the medical student clerkship director and the HSD Pediatric residency director. Manuals that included a primer on case-based learning for facilitators and topic areas of neonatal fever, cough, diarrhea, and seizure were created. In early analysis, all involved learners found the session helpful and enjoyable; citing developing differential diagnoses, collaborative learning, and clinical reasoning as major learning themes (5). Another example of training that has been successful is the annual resuscitation workshop, based on the PALS curriculum, which has always elicited a positive reception from the HSD staff.

One partner site, with an emergency medicine subspecialist lead, also provides an annual procedural skills workshop. This workshop trains HSD-HBM residents in practical skills such as suturing, intubation, and chest tube placement. The importance of these workshops cannot be overstated, as residents often put these skills into practice that very same week. These initiatives mold and adapt at the request and expressed needs of HSD and over the last 3 years have included more involvement with directly teaching Haitian residents. The HSD-HBM residency leadership has also encouraged residents to present lectures, often anchored to a particular challenging case, at the National Haitian Pediatric Congress; providing public-speaking and leadership experience for these residents. In addition, SDC was able to support the printing of HSD-HMB senior pediatric residents’ posters of case reports, quality improvement, and research projects for the 2016 National Haitian Pediatric Congress.

Most importantly, the collaborative has also committed to bidirectional exchange, providing each senior resident at HSD with the opportunity to participate in 4-week rotations at SDC partner sites in NA, with the goal of obtaining experience in more technologically advanced subspecialties, not yet as developed in Haiti. In 2016, five of the six senior HSD-HMB residents traveled to partner sites and completed observerships in cardiology and pediatric intensive care. The Haitian residents rated this experience very highly, stating that the opportunity allowed them to gain clinical skills that help them better care for patients, become better educators, and strengthen their sense of partnership with members of SDC. In addition, the members of SDC have obtained funding and provided opportunities for a number of the HSD faculty and staff, including attending physicians, nurses, radiology technicians, pharmacists, microbiology technicians, and hospital administrators, to participate in experiences and other training not available in Haiti. Visiting residents to Haiti are exposed to clinical cases and acuity not often seen in NA, but common in a resource-limited setting (RLS). Upon return, visiting residents give case presentations to their home institutions, further embedding global health indicators in their curriculum and allowing others to benefit. In addition, many of the NA partner sites ask that the returning visiting residents engage in ongoing fundraising efforts for HSD.

### Research

One SDC partner site offers a funded 3-week intensive research training program that allows researchers from RLS to attend at no cost. Seven Haitian healthcare professionals, four from HSD, have successfully completed the advanced research training. This is a small step toward building an infrastructure that can bring measurement and evaluation science to HSD. In addition, this partner site allows for its own trainees and faculty to apply for seed funding to support small research projects that provide mutually beneficial data and advance research capacity (6). Another partner site began a large prospective randomized study evaluating the feasibility of hydroxyurea in sickle cell disease. This project enrolled 43 participants in 2016 and is actively collecting data. Hydroxyurea has limited data for use in RLS, yet holds potential for cost savings and improved clinical outcomes that could improve sickle cell care in Haiti. Measurement and evaluation of interventions will be essential to identify best practices in Haiti and RLS and as a priority also reflects the SDC commitment to transparency and responsibility to the field of global health.

## Challenges

The SDC was developed primarily to facilitate coordination of visiting faculty and thus target a common challenge in global health, specifically: how to help without hurting. HSD was clear that large groups were disruptive and that long-term relationships seeking to find common goals were favored over short-term relief missions. Early visits from partner sites were not always well structured, resulting in confusion for the supervising Haitian attending in delegating clinical responsibility to visiting staff. This also made it difficult for visiting residents to define their role in caring for patients. The coordination provided by the SDC leadership helped to identify specific roles that were more clearly defined. One example is partnering HSD-HMB residents with visiting residents in the emergency department with a Haitian attending overseeing their efforts. In this model, residents work together to provide patient care, and in the process, share medical knowledge as well as information about culture and systems. Another example is the practice of posting a welcome sign for each visiting group which both welcomes and clarifies the partnership. This seems to help with expectations of reciprocity. While political unrest in Haiti has prevented groups from traveling, it has been institutional specific and beyond control of the collaborative. During one visit, protests and roadblocks prevented Haitian staff from getting to HSD. The visiting team, staying in housing close to the hospital, was able to staff the hospital in their absence. In the past, coordination in Haiti has fallen heavily on the shoulders of the Medical Director and CEO. The addition of a residency coordinator at HSD and support for a chief resident has defrayed this responsibility, allowing the CEO and Medical Director to address more pressing issues. Developments such as these have been supported directly from SDC fundraising and build on successful identification of needs from both sides.

## Conclusion

The 5-year collaborative effort between NA-AMCs with Haiti’s only freestanding children’s hospital described here provides an example of an ethically responsible academic medical collaborative with efforts that are sustainable, focused on building local capacity, and driven toward meeting the expressed needs of HSD. SDC provides a potential model for other institutions wishing to transition from immediate disaster response to long-term partnerships.

A recent shift in the approach of AMCs from single site projects reserved for participation by a single institution to multi-institutional collaboratives and partnerships has been observed (7–9). An advantage of this model is coordination of efforts among AMCs to ensure continuity of services and support while attempting to minimize overlap and redundancies. It is often a challenge to maintain exclusive bidirectional partnerships except for those from very large AMCs. Small to medium size AMCs and residency programs may struggle to maintain continuity of services and support in exclusive bidirectional partnerships. The model presented here allows resident physicians from numerous AMCs of all sizes to experience a partnership in an RLS. A disadvantage of this model is that the majority of trips to Haiti are for a month or less due to time constraints of visiting faculty and resident physicians. Other bidirectional partnerships such as SEED Global Health, and the Rwanda Human Resources for Health program have emphasized longer term commitments (1 year or longer). In the case of Haiti, it is hoped that some of the disadvantages of shorter term personal commitments are overcome by scope of the project and the close coordination of the members, which allow for a nearly continuous presence at HSD.

There are several unique features of SDC when compared to similar AMCs:

- Focus on supporting the training of local physicians
- Bidirectional exchange of residents, faculty, and staff
- Combination of small, medium, and large residency programs
- Nearly continuous presence of staff and host institution
- Largely self-funded
- Annual fundraising efforts at partner sites to directly support host institution.

This collaborative was born out of an effort to create a sustained partnership with Haiti after the immediate international response to the 2010 earthquake. Unlike the program in Rwanda, our efforts have largely been self-funded through privately organized fundraisers with little financial investment from home institutions; however, our model demonstrates that when AMCs combine efforts, much can be done with minimal expense. Another important feature of our collaborative is the bidirectional exchange model, where we successfully invited Haitian pediatric resident physicians from HSD to participate in month-long electives at AMCs in NA. The benefits of intercultural learning and wider clinical exposures are win-wins on both sides, but metrics to quantify these benefits are needed to support funding and grant requests.

Academic medical centers seeking to become involved in efforts to improve global health in RLS should strongly consider putting their energies toward collaboratives to increase the potential impact and to develop programs in a mutually beneficial and sustainable way. Increasing the capacity to educate healthcare workers should be a priority for these partnerships since the shortage of healthcare professionals is the root cause of health disparities in the world.

## Author Contributions

All authors participated in all aspects of planning and drafting of this manuscript. Each author independently participated in the events described and are active members of the collaborative. MK and JW had equal efforts as first authors in the initial draft, though all authors provided input, edits, and approved the final manuscript as submitted.

## Conflict of Interest Statement

The authors declare that the research was conducted in the absence of any commercial or financial relationships that could be construed as a potential conflict of interest.

## Abbreviations

AMC, academic medical center; CPR, cardiopulmonary resuscitation; HSD, Hôpital Saint Damien-Nos Petits Frères et Soeurs; HBM, Hôpital Bernard Mevs; NA, North America; NGO, Non-Government Organization; PALS, pediatric advanced life support; RLS, resource-limited setting; SDC, Saint Damien Academic Medical Collaborative; USAID, United States Agency for International Development.
